# Study on the influence of traveling wave effect on Γ-type combined structure using equivalent simplified model

**DOI:** 10.1371/journal.pone.0289471

**Published:** 2023-08-01

**Authors:** Laite Sun, Yu Bai, Zhengcong Lai

**Affiliations:** 1 Faculty of Civil Engineering and Mechanics, Kunming University of Science and Technology, Kunming, Yunnan, China; 2 Earthquake Engineering Researching Center of Yunnan, Kunming University of Science and Technology, Kunming, Yunnan, China; University of Sharjah, UNITED ARAB EMIRATES

## Abstract

The Γ-type combined structure is an unconventional high-rise long-span structure, and there is no research literature about the influence of traveling wave effect on such structure at present. This study established an accuracy equivalent simplified model for such structure, employed the modified large mass method to carry out a time history analysis on the seismic response such as the shear force, axial force, and bending moment of the model affected by the traveling wave effect, and summarized the corresponding laws through the analysis results of different working conditions such as time difference, mass ratio, and stiffness ratio. The results demonstrate that, with the increase in the input time difference of seismic wave, the bending moment of the control section increases gradually, while the shear force and axial force decrease first and then increase, and traveling wave effect response within a certain range of time difference is weaker than the uniform excitation response. The differences of traveling wave effect responses due to the change of mass ratio between the vertical and horizontal parts of the structure are less than 10%, while the differences due to the change of stiffness ratio are greater than 20%.

## 1. Introduction

With the progress of architectural design level and the development of construction technology [1, 2], the buildings gradually develop in the direction of high (towering structure, high-rise structure) and large (large span structure, large space structure), and a large number of super high-rise long-span landmark buildings emerge around the world, such as CCTV headquarters (Beijing), Marina Bay Sands Hotel (Singapore), Umeda Sky Building (Osaka) and La Défense Gate (Paris). For super high-rise structures, the effects of horizontal loads such as seismic loads usually dominate the structural design, and super high-rise long-span structures additionally need to consider the effects of spatial vibrations of ground motion such as traveling wave effects.

Since the propagation speed of seismic waves is a finite value, a time delay occurs when they reach different supports of the structure, i.e., the traveling wave effect. For long-spans or structures with long delay times, the effect of traveling waves on the seismic response cannot be ignored. Léger [3] used the relative motion method and the large mass method to calculate the seismic response of a four-span bridge under the influence of traveling wave effects, showing that the structural response tends to increase as the wave velocity decreases and can become significantly larger than the response obtained from synchronous excitation. Heredia-Zavoni [4] investigated the traveling wave effect in three-dimensional, multi-storey, multi-span, symmetric, linear elastic buildings and found that the traveling wave effect plays a larger role for columns in the ground level of stiff systems. Zhang [5] combined the pseudo-excitation method and the complex mode superposition method to give the closed solution of the smooth random seismic response of the long-span structure considering the traveling wave effect, and the results show that the traveling wave effect can have a significant effect on the response of the long-span structure. Meng [6] developed a three-dimensional nonlinear finite element model of a high-pier bridge and analyzed the differences between uniform excitation and multi-support excitation considering traveling wave effects, demonstrating that traveling wave effects amplifies seismic pounding response, while the ends of piers are adversely affected, exacerbating the damage, and which has obvious influences to all bearings. Dai [7] clarified the seismic response law of acceleration and strain of buried oil and gas pipeline under bidirectional multi-point excitation through experiments, and the results show that the curve of soil acceleration amplification coefficient under bidirectional multi-point excitation has a wider fluctuation range, and the soil displacement changes more significantly compareing with the bidirectional consistent excitation.

From the aforementioned and other research results [8–12], it can be seen that the current research method on traveling wave effect is basically to establish a finite element model for a specific structure for numerical simulation, and the obtained research results are only valid for a specific research object, but cannot form a universal law and apply to other structures with the same characteristics. In addition, the current research on traveling wave effect is mainly concentrated in common structure types such as bridges, long-span space structures and dams, and there are relatively few studies on the influence of traveling wave effect on super high-rise long-span structures.

**Fig 1:**
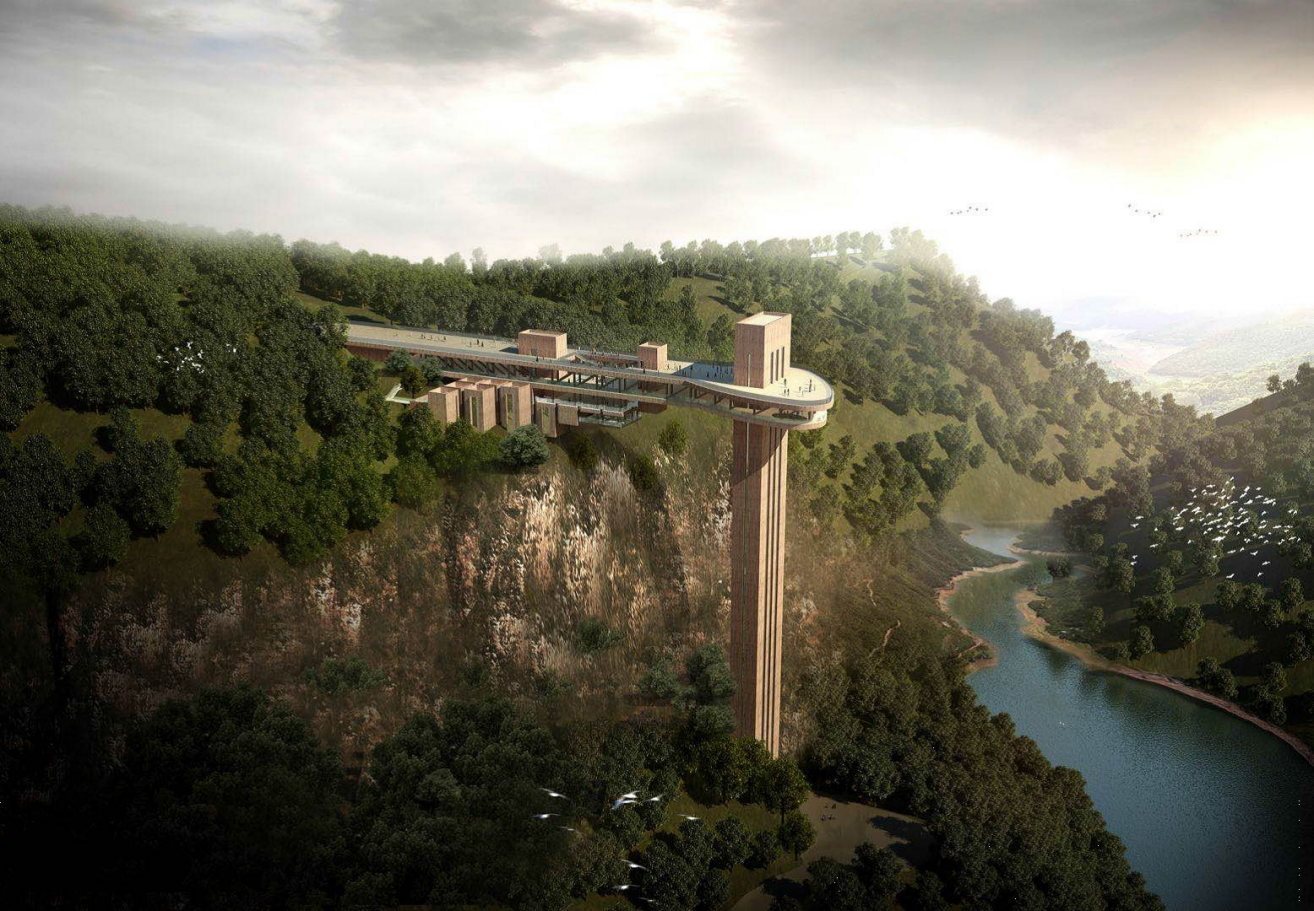
A Γ-type combined structure.

The Γ-type combined structure, as a special super high-rise long-span structure combined by two different structural forms, is extremely rare in the engineering world (Fig 1). The authors have reviewed a large number of research literature and engineering cases, and no research results on the issue of such structures affected by traveling wave effect have been found.

To address the limitations of the existing research method of traveling wave effect and the gap in the research field of seismic response of Γ-type combined structure, this paper proposes to establish an equivalent simplified model to simulate the Γ-type combined structure. In the basic theoretical study of structural dynamics [13], the idea of equivalent simplification is always present in both single-degree-of-freedom and multi-degree-of-freedom systems, and the classical vertical tandem multi-degree-of-freedom model is the most typical equivalent simplified model. In order to study specific structural types or special complex structures, researchers often establish corresponding equivalent simplified models according to the structural characteristics, and determine the dynamic properties of the original structure by studying the model under the premise of ensuring the accuracy of the model, so as to simplify the complex problems. Li [14] regarded tall buildings and high-rise structures as a cantilever bar with variable cross-section when analysing free vibrations of such structures, and established the differential equations of free longitudinal vibrations of bars with variably distributed mass and stiffness considering damping effect. Bilello [15] investigated the dynamic response of a small-scale bridge model under a moving mass based on the continuous Euler–Bernoulli beam theory, and validated the analytical solution through a series of experiments. Hu [16] presented a novel model conversion technique for structural dynamic systems that is capable of converting a physically realizable, higher-order model termed the source model into a completely different, predefined, physically realizable, lower-order model termed the target model. Huergo [17] developed an equivalent coupled-two-beam discrete model for time‐domain dynamic analysis of high‐rise buildings with flexible base and carrying any number of tuned mass dampers, and applied the equivalent model to a shear wall–frame building located in the Valley of Mexico. Casagrande [18] presents a simple yet robust frame model to be used as an alternative to continuous 2D or 3D finite element models for the purpose of analyzing multi-storey cross-laminated timber shearwalls with openings when subjected to lateral loads, pushover numerical analyses were conducted on the proposed equivalent frame model and 2D finite element model, and the results showed good agreement between the two models along the entire force-displacement curves.

This paper establishes an equivalent simplified model with full consideration of the structural characteristics of the Γ-type combined structure, verifies the accuracy of the model by comparing it with the finite element model, sets up a series of representative working conditions from the basic formula for calculating the seismic response of multi-point excitation by using the large mass method, summarized the corresponding laws of the influence of traveling wave effect on the seismic response of the structure through the analysis results.

## 2. Establishment of equivalent simplified model

The Γ-type combined structure is a combination of vertical shear wall structure and horizontal steel truss structure. Since the two kinds of structure are very different, the vertical part and the horizontal part of the Γ-type combined structure should be treated separately when performing the equivalent simplification. Firstly, many scholars have conducted a lot of researches on how to simplify the shear wall structure equivalently, they simulated shear wall structures using an equivalent cantilever beam model based on the assumption of continuous connection links, and verified the accuracy of this method through finite element method [19–21]. According to the aforementioned literature, the equivalent beam model was established for the vertical part of the Γ-type combined. Secondly, the traditional lumped-mass shear model is applicable to steel truss structure because of the large differences in mass and stiffness distribution along the length of such structure [22].

**Fig 2:**
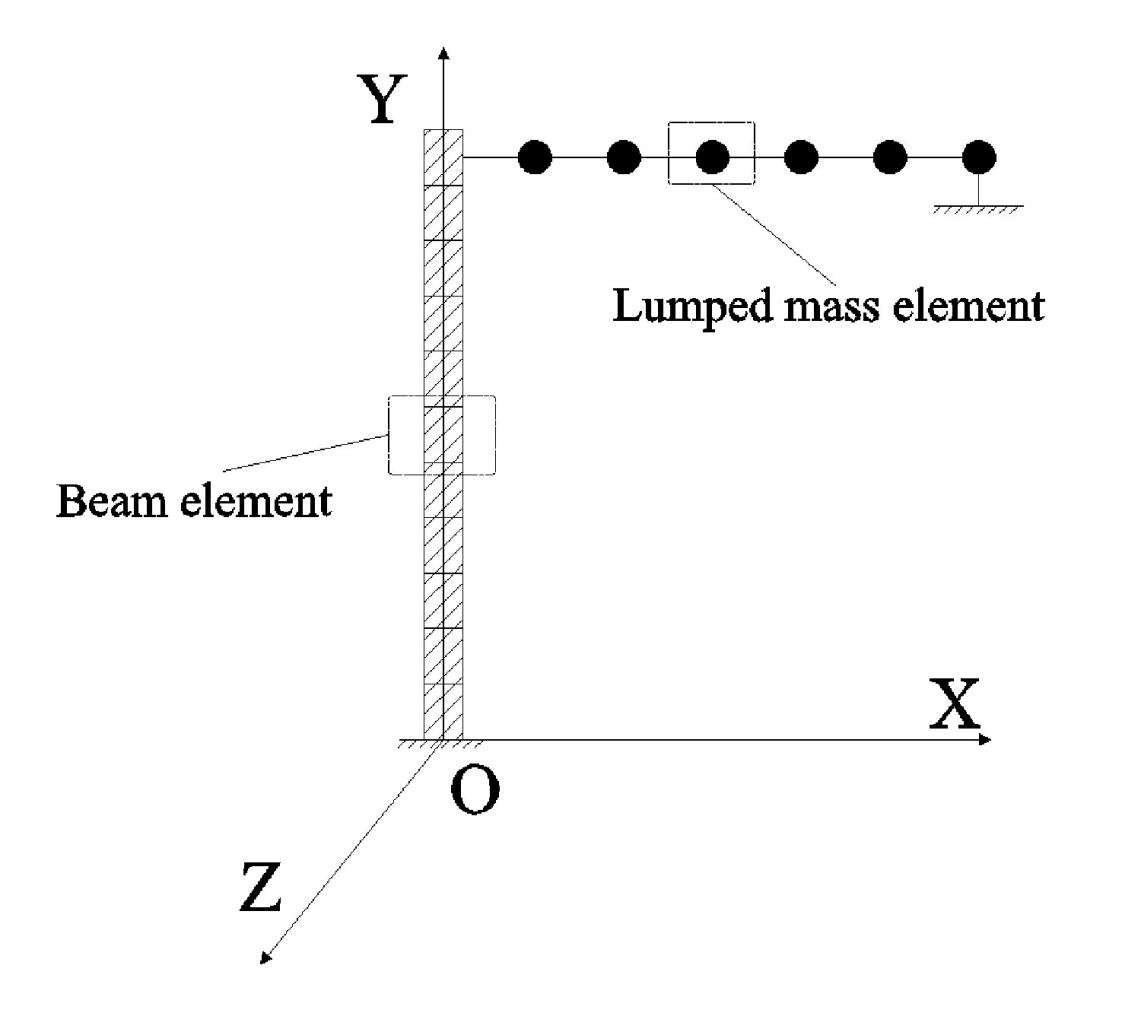
Equivalent simplified model.

The simplified model shown in Fig 2 was equivalent to the Γ-type combined structure. The global rectangular coordinate system was established at the bottom center of the vertical part of the model as the origin, and then the local rectangular coordinate system was established at the center of the end of the vertical and the horizontal part as the origin.:

According to the Timoshenko beam theory, the stiffness matrix of the beam element under the influence of shear force and bending moment considering the shear deformation is as follows [23]:

$$\left[k_{B}^{e}]=\frac{EI}{L^{3}(1+\varphi )}[\begin{array}{cccc} 12 & 6L & -12 & 6L\\ 6L & (4+\varphi )L^{2} & -6L & (2-\varphi )L^{2}\\ -12 & -6L & 12 & -6L\\ 6L & (2-\varphi )L^{2} & -6L & (4+\varphi )L^{2} \end{array}\right]$$ (1)

Where E is the elasticity modulus of materials, I is the equivalent moment of inertia of the element section, and L is the unit length. φ = 12EI/A_S_GL^2^, where A_S_ is the unit shear area, and G is the shear modulus of materials.

Consistent mass matrix of the beam element:

$$\left[m_{B}^{e}]=\frac{\rho AL}{420}[\begin{array}{cccc} 156 & 22L & 54 & -13L\\ 22L & 4L^{2} & 13L & -3L^{2}\\ 54 & 13L & 156 & -22L\\ -13L & -3L^{2} & -22L & 4L^{2} \end{array}\right]$$ (2)

Where ρ is the mass density of materials, and A is the equivalent unit cross-sectional area.

According to the association between the local and global coordinate system of the beam element, the transition matrix [T^e^]is defined as:

$$\left[T^{e}]=[\begin{array}{cccc} \cos\theta & 0 & 0 & 0\\ 0 & 1 & 0 & 0\\ 0 & 0 & \cos\theta & 0\\ 0 & 0 & 0 & 1 \end{array}\right]$$ (3)

Where θ is the included angle between the local and global coordinate system of the beam element, θ = 90° in this model.

Global coordinate stiffness matrix of beam element:

$$\left[k_{B}^{eT}]={\left[T^{e}\right]}^{T}[k_{B}^{e}][T^{e}\right]$$ (4)

Global coordinate mass matrix of beam element:

$$\left[m_{B}^{eT}]={\left[T^{e}\right]}^{T}[m_{B}^{e}][T^{e}\right]$$ (5)

According to the actual conditions and research needs, the beam model can be discrete into a limited number of beam elements. The stiffness matrix[K_B_] and mass matrix[M_B_] of the beam model can be assembled from the corresponding matrices of these beam elements through the direct superposition method.

For the lumped mass model, the stiffness matrix[K_M_] under the influence of shear force and bending moment can be determined by the direct equalization method based on the definition of stiffness influence coefficient. The mass matrix[M_M_] is a diagonal matrix, with the mass concentrated in the translational degrees of freedom and the rotational degrees of freedom being zero. The overall structural stiffness matrix[K] is composed of the beam model’s stiffness matrix[K_B_] and the lumped mass model’s stiffness matrix[K_M_], and so is the overall structural mass matrix[M].

The structure’s natural frequency ω and its mode can be obtained by solving the characteristic equation |[K]-ω^2^[M]| = 0 based on the boundary condition that the structure is fixedly connected with the structural bearings. Since the characteristic equation is complex and difficult to obtain an analytical solution, software programming can be employed to obtain the numerical solution.

The structure adopts the Rayleigh damping, and the corresponding matrix[C] is:

[C]=α[M]+β[K] (6)

$$\begin{array}{l} \alpha =\frac{2\omega_{1}\omega_{2}}{\omega_{2}^{2}-\omega_{1}^{2}}(\omega_{2}\zeta_{1}-\omega_{1}\zeta_{2})\\ \beta =\frac{2}{\omega_{2}^{2}-\omega_{1}^{2}}(\omega_{2}\zeta_{2}-\omega_{1}\zeta_{1}) \end{array}$$ (7)

Where ω_1_ and ω_2_ are the first second-order frequencies of the structure, and ζ_1_ and ζ_2_ are the structural damping ratio, all taken according to the actual conditions.

## 3. Verification of equivalent simplified model

### 3.1 Model establishment

In order to verify the accuracy of the equivalent simplified model for the simulation of Γ-type combined structure, the cliff hotel shown in Fig 2 is selected as the research object in this paper, and the equivalent simplified model and the finite element model of the building are established respectively, and the errors between the two models are analyzed through the comparison of vibration mode diagram, natural vibration period, and the vertex displacement time history.

**Fig 3:**
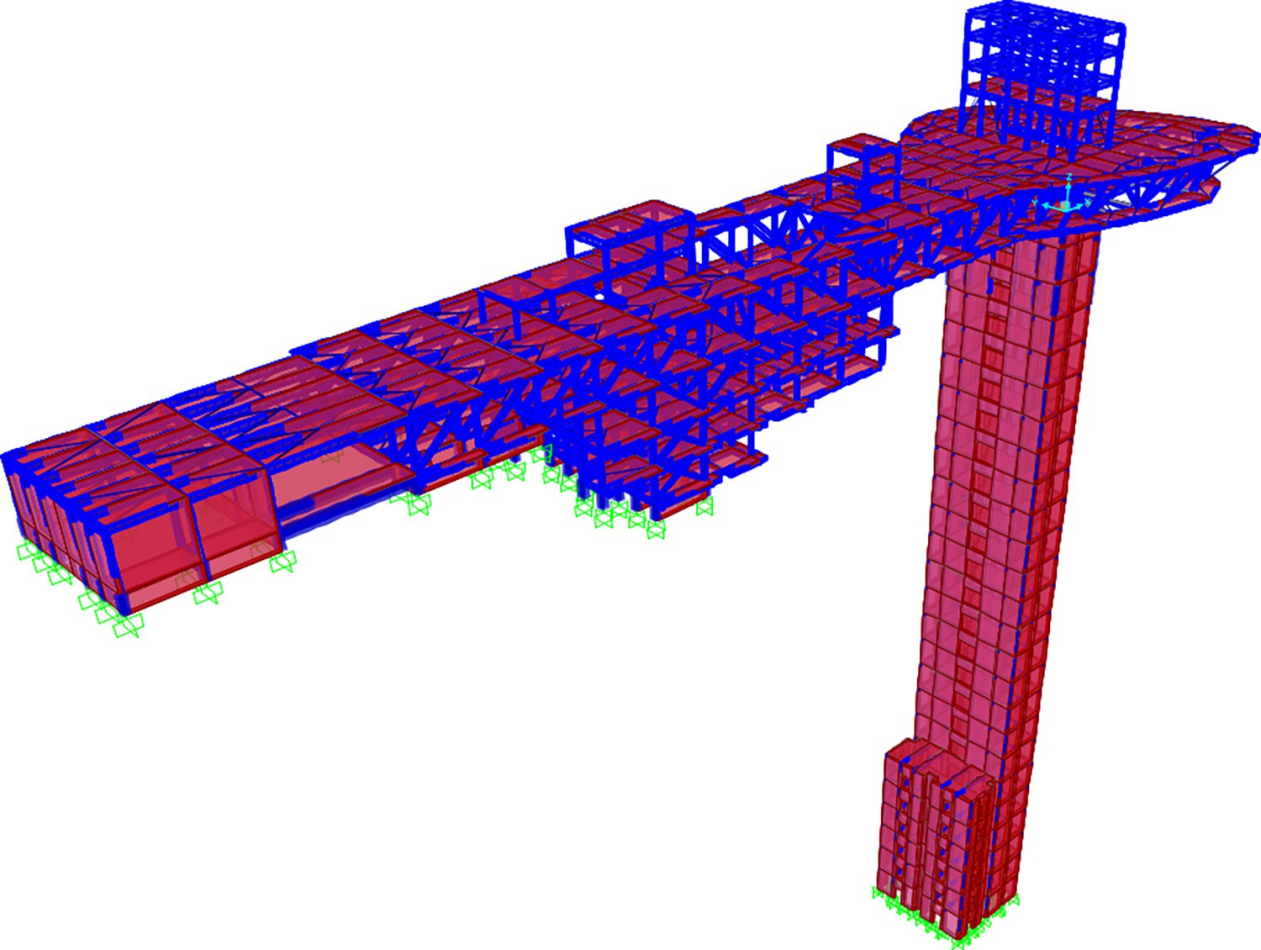
Finite element model.

The research object consists of a high-rise shear wall structure as a sightseeing elevator and a long-span steel truss structure as a scenic hotel. The height above the ground (H_0_) of the building’s reinforced concrete shear wall is 135 m, the mass of the shear wall (M_H_) is 21760 t, the length of the steel truss (L_0_) is 152.7 m, the maximum span (L_S_) is 70.5 m, and the mass of the steel truss (M_L_) is 17516 t. To properly model the structure, the frame element is used for beams and columns, the thin shell element is used for slabs, and the nonlinear multi-layer shell element is used for shear walls. When meshing, the shape and size of the mesh are automatically adjusted through FEM software to match the nodes inside the element. Concrete C30 was adopted for plates and beams, and C60 was adopted for shear wall and column. The normal rebar was employed with HPB335 and HRB400, and profile steel was employed with Q355. The finite element model of the structure was established in the software as shown in Fig 3.

Firstly, this study made a simple static analysis of the finite element model of the structure to obtain the equivalent stiffness EI_equ_ (Yu and Zhang 2009). Following the common practice in engineering, this study utilized the uniformly distributed horizontal lateral load pattern for analysis, based on the equivalent principle that the horizontal displacements of the structure vertices are equal. After applying uniformly distributed horizontal load to the shear wall along the height in the X-axis direction of the structural model, the total shear force P_0_ at the bottom was 20570 kN, and the horizontal displacement Δ_X_ of the floor centroid at the height of 135 m was calculated to be 65.42 mm. Then the equivalent load was applied to the equivalent simplified model, and the equivalent section moment of inertia I_equ_ was calculated to be 5.126×1013 mm^4^ under the condition of the displacement in the X-axis direction at the height of 135 m δ_X_ = Δ_X_.

Secondly, the vertical part and the horizontal part of the equivalent simplified model were discretized into several elements according to the structural information. The vertical part was divided into beam elements based on the floors of the shear wall structure, and the length and mass were evenly distributed to each element. The horizontal part was divided into lumped mass elements according to the steel truss structure axis network, and the mass and connection length of each element were determined according to the grid size and the total mass of the components in each grid.

### 3.2 Model comparison

**Fig 4:**
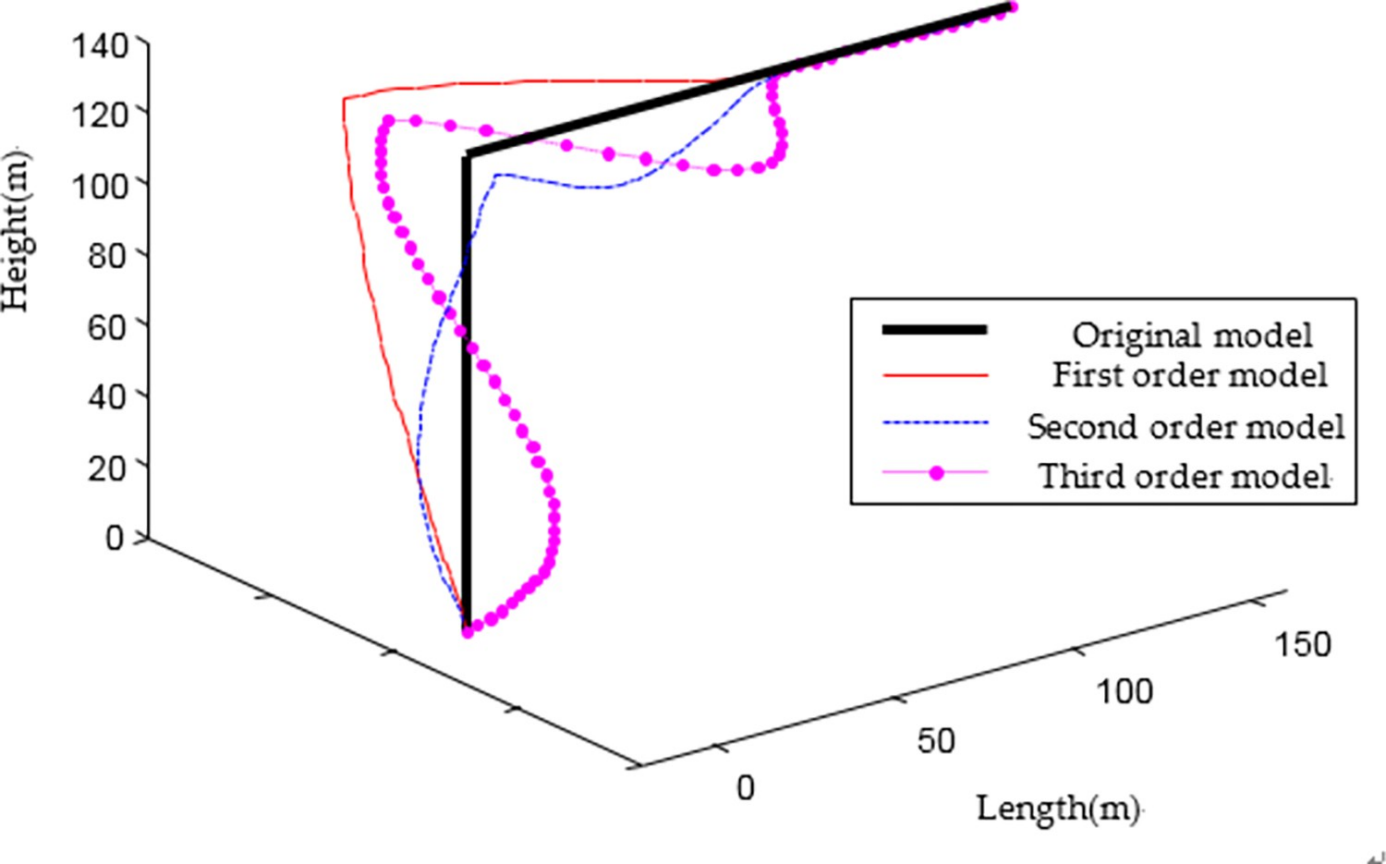
Vibration mode diagram of equivalent simplified model.

**Fig 5:**
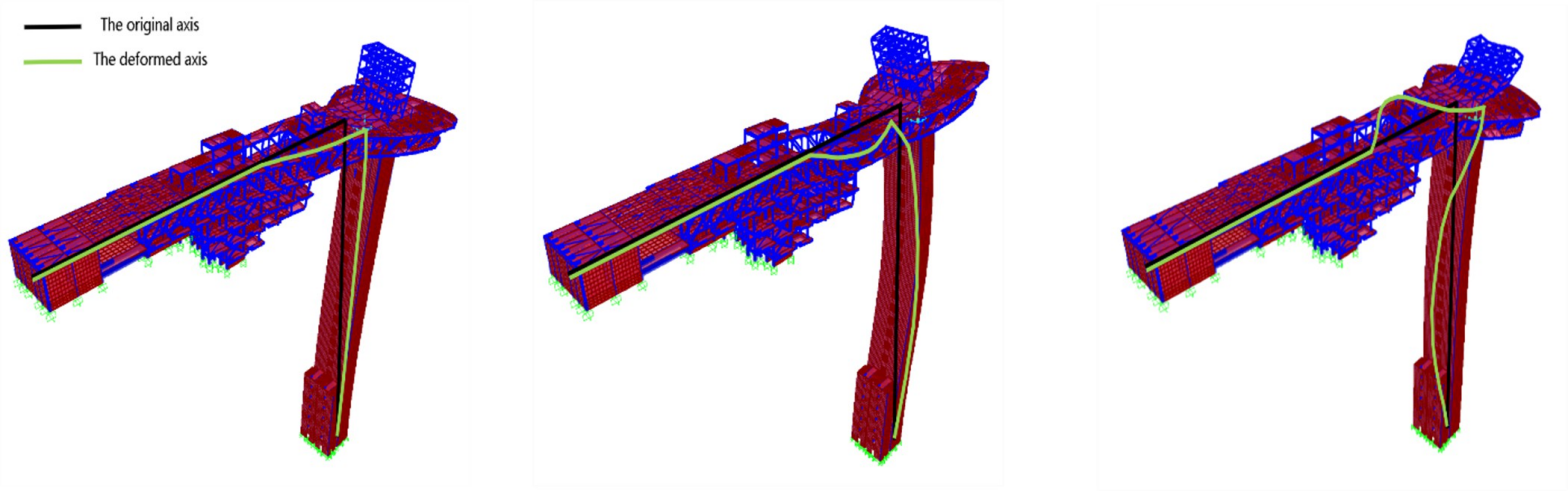
Vibration mode diagram of finite element model. (a) 1st order model; (b) 2nd order model; (c) 3rd order model.

The equivalent parameters of each element are substituted into the characteristic equation, and the first triplet of vibration modes of the equivalent simplified model is obtained by solving the equation (Fig 4), which is basically consistent with the three-dimensional mode diagram obtained by the finite element model (Fig 5).

**Fig 6:**
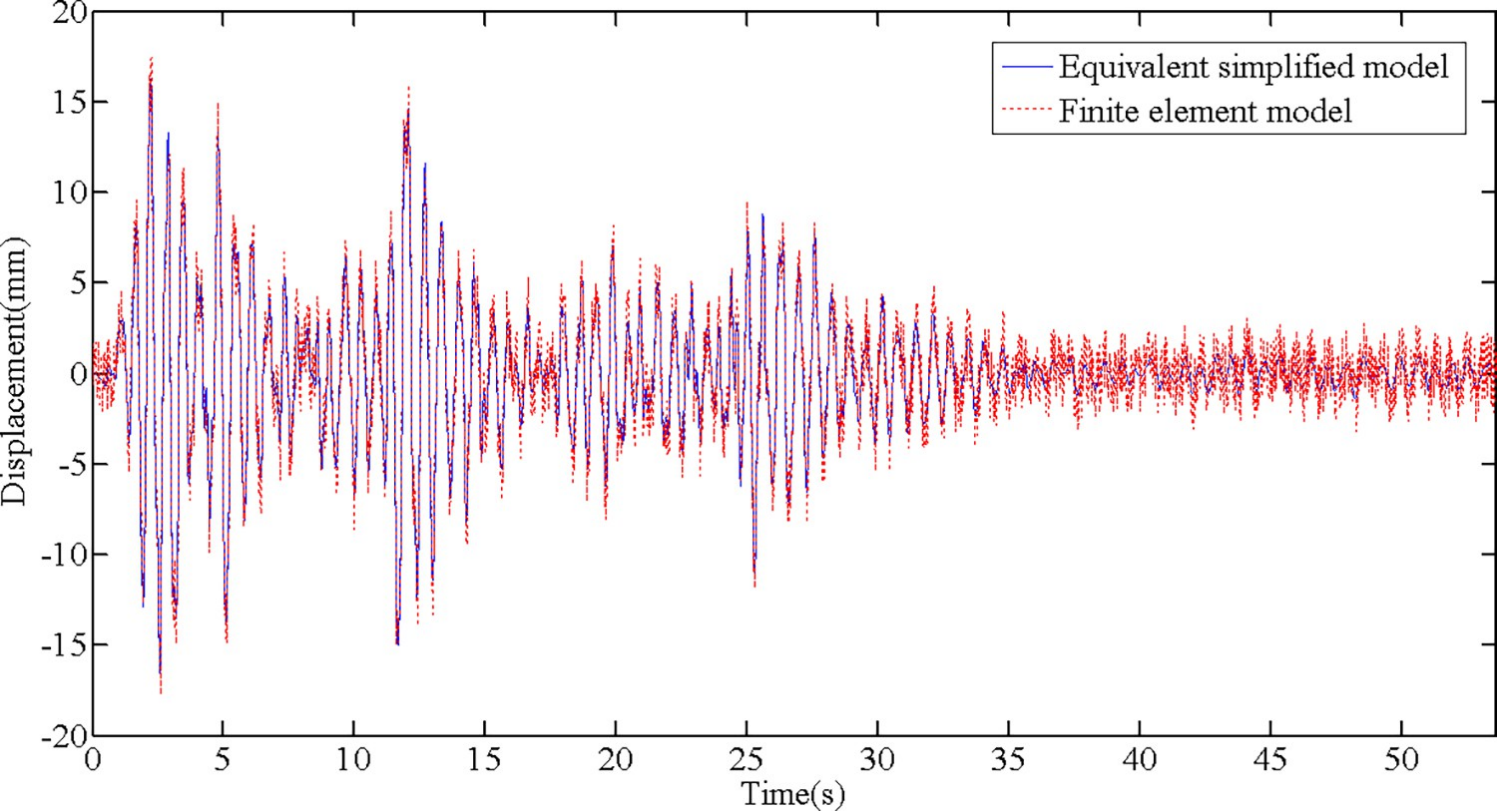
Comparison of vertex displacement time history between equivalent simplified model and finite element model.

The first triplet of the natural vibration period of the equivalent simplified model is displayed in Table 1. Through data comparison, the natural vibration period calculated using equivalent parameters is approximately the same as that of the finite element model, and the maximum deviation is only 5.84%. As shown in Fig 6, by inputting the same artificial wave for time history analysis, the displacement time history curves of the equivalent simplified model and finite element model at the same point on the structure vertex are consistent.

**Table 1:** Natural vibration period.

	Finite element model T/s	Equivalent simplified mode T/s	Deviatio %
1^st^ order model	1.6070	1.7008	5.84
2^nd^ order model	0.6440	0.6574	2.08
3^rd^ order model	0.5049	0.5293	4.83

Through the comparison between the equivalent simplified model and finite element model in the first triplet of vibration mode diagram, natural vibration period, and the vertex displacement time history, it can be concluded that the equivalent simplified model can effectively simulate Γ-type combined structure, with accurate calculation results, which can be used for general law research.

## 4. Calculation method of seismic response considering traveling wave effect

For general structures that do not have to consider traveling wave effects, the calculation method of uniform excitation is used to calculate the seismic response:

$$\left[M]\{\ddot{X}\}+[C]\{\dot{X}\}+[K]\{X\}=\{P\right\}$$ (8)

As for the seismic response of non-uniform excitation, when subjected to non-uniform excitation input from b structural bearings, the motion differential equation of multi-degree-of-freedom linear structure with a degree of freedom s can be expressed by the following block matrix [13]:

$$\left[\begin{array}{cc} M_{ss} & M_{sb}\\ M_{sb}^{T} & M_{bb} \end{array}]\{\begin{array}{c} {\ddot{X}}_{s}\\ {\ddot{X}}_{b} \end{array}\}+[\begin{array}{cc} C_{ss} & C_{sb}\\ C_{sb}^{T} & C_{bb} \end{array}]\{\begin{array}{c} {\dot{X}}_{s}\\ {\dot{X}}_{b} \end{array}\}+[\begin{array}{cc} K_{ss} & K_{sb}\\ K_{sb}^{T} & K_{bb} \end{array}]\{\begin{array}{c} X_{s}\\ X_{b} \end{array}\}=\{\begin{array}{c} 0\\ R_{b} \end{array}\right\}$$ (9)

Where X_s_ is the displacement vector of the structure; X_b_ is the displacement vector of the structural bearings; R_b_ is the force of ground motion acting on the bearing points. The matrix with subscript s represents the matrix related to the non-bearing points, and the matrix with subscript b represents the matrix related to the bearing points.

At present, there are two main methods to solve the non-uniform excitation seismic response: time-domain methods and frequency-domain methods. The time-domain method can directly reflect the transmitting characteristics of seismic motion using the time difference/phase difference, occupying a dominant position in practical applications due to its clear calculation principle. Time domain methods generally include the direct solving method, displacement method, relative motion method, large mass method, etc. [22–24]. The large mass method is a method to solve the calculation through the equivalent of the structure’s model from the perspective of mechanics, which is easy to realize in the general finite element analysis software. The large mass method can be utilized to calculate the structural response conveniently and quickly, and the results obtained are similar to the actual total seismic response of the structure.

### 4.1 Large mass method

By releasing the constraint along the seismic excitation direction, the large mass method can increase the large mass ML with a rigid connection to the structure and the seismic acceleration time so as to apply the equivalent load M_L_Ü_0_ on the large mass and indirectly transmits it to the structure. Generally, the weight of the large mass is 105 to 108 times the total weight of the structure.

The supporting reaction force is equal to the product of the bearing mass and the ground acceleration. The second term of Formula (9) is expanded as:

$$M_{sb}^{T}{\ddot{X}}_{s}+M_{L}{\ddot{X}}_{b}+C_{sb}^{T}{\dot{X}}_{s}+C_{bb}{\dot{X}}_{b}+K_{sb}^{T}X_{s}+K_{bb}X_{b}=M_{L}{\ddot{U}}_{0}$$ (10)

Since M_L_ exceeds other values by several orders of magnitude, the above formula can be simplified as follows:

$${\ddot{X}}_{b}\approx {\ddot{U}}_{0}$$ (11)

### 4.2 Modified large mass method

In practical applications, when the large mass method is applied in the seismic response analysis of the structure, if Rayleigh damping is used, the calculation results may be greatly deviated [25]. The reason is that large mass nodes will lead to very large mass damping of the structure, and Formula (10) will be simplified to Formula (12):

$${\ddot{X}}_{b}+M_{L}^{-1}C_{bb}{\dot{X}}_{b}\approx {\ddot{U}}_{0}$$ (12)

If Rayleigh damping is used, then C_bb_ = α•M_L_+β•K_bb_, Formula (12) can be simplified as:

$${\ddot{X}}_{b}+\alpha {\dot{X}}_{b}\approx {\ddot{U}}_{0}$$ (13)

Since the coefficient α is not an infinitesimal quantity, Eq (11) no longer holds, and the larger the coefficient α is, the larger the error is.

If Eq (11) holds, Eq (13) can be modified as:

$${\ddot{X}}_{b,real}={\ddot{U}}_{0}+\alpha {\dot{U}}_{0}$$ (14)

Where Ẍ_b,real_ is the real input seismic acceleration of the large mass, and α is the mass correlation coefficient of Rayleigh damping obtained by Eq (7).

## 5. Calculation of seismic response of Γ-type combined structure under the influence of traveling wave effect

When calculating the seismic dynamic response, the traveling wave effect can be reflected by the time difference of seismic wave input at different structural bearings. The Γ-type combined structure discussed in this study is a combination of two different structural forms, and according to Formula (9), the mass and stiffness differences existing between its vertical and horizontal parts can significantly affect the seismic dynamic response of the structure. In order to study the effect of traveling wave effect on the seismic response of Γ-type composite structure, an equivalent simplified model is established according to the method described in the previous section, and the modified large mass method is applied to analyze the model in time history under different working conditions from the time difference of seismic wave input at different structural bearings and the mass and stiffness differences existing between its vertical and horizontal parts.

### 5.1 Model initial parameter setting

Firstly, it is assumed that the mass and stiffness of the vertical part of the complex structure are uniformly distributed along the height, the mass of the horizontal part is evenly distributed at each lumped mass point, and the stiffness is evenly distributed along the length. Secondly, it is assumed that the total mass, stiffness, and other basic parameters of the vertical and horizontal parts conform to a certain proportional relationship. The mass ratio coefficient Г_m_ is defined as M_L_/M_H_, where M_L_ refers to the total mass of the horizontal part, and M_H_ refers to the total mass of the vertical part. The stiffness ratio coefficient Г_k_ is defined as K_L_/K_H_, where K_L_ represents the flexural stiffness of the horizontal part, and K_H_ represents the flexural stiffness of the vertical part.

The initial setting of the equivalent simplified model is that Г_m_ = 1.0 and Г_k_ = 1.0. Specifically, for the vertical part, the height H is 100 m, and the cross-sectional area of the beam element is 5 m×5 m. The elasticity modulus of materials is 32.5 MPa, and the material density is 2550 kg/m^3^. As for the horizontal part, the length L is 100 m, and the part is divided into 10 lumped mass units, with a mass of 637.5t for each unit. The seismic fortification intensity of the model is set as 8 degrees (0.2g), the site predominant period T_g_ is 0.45s, and the maximum horizontal seismic coefficient α_max_ is 0.16.

### 5.2 Seismic wave selection

**Fig 7:**
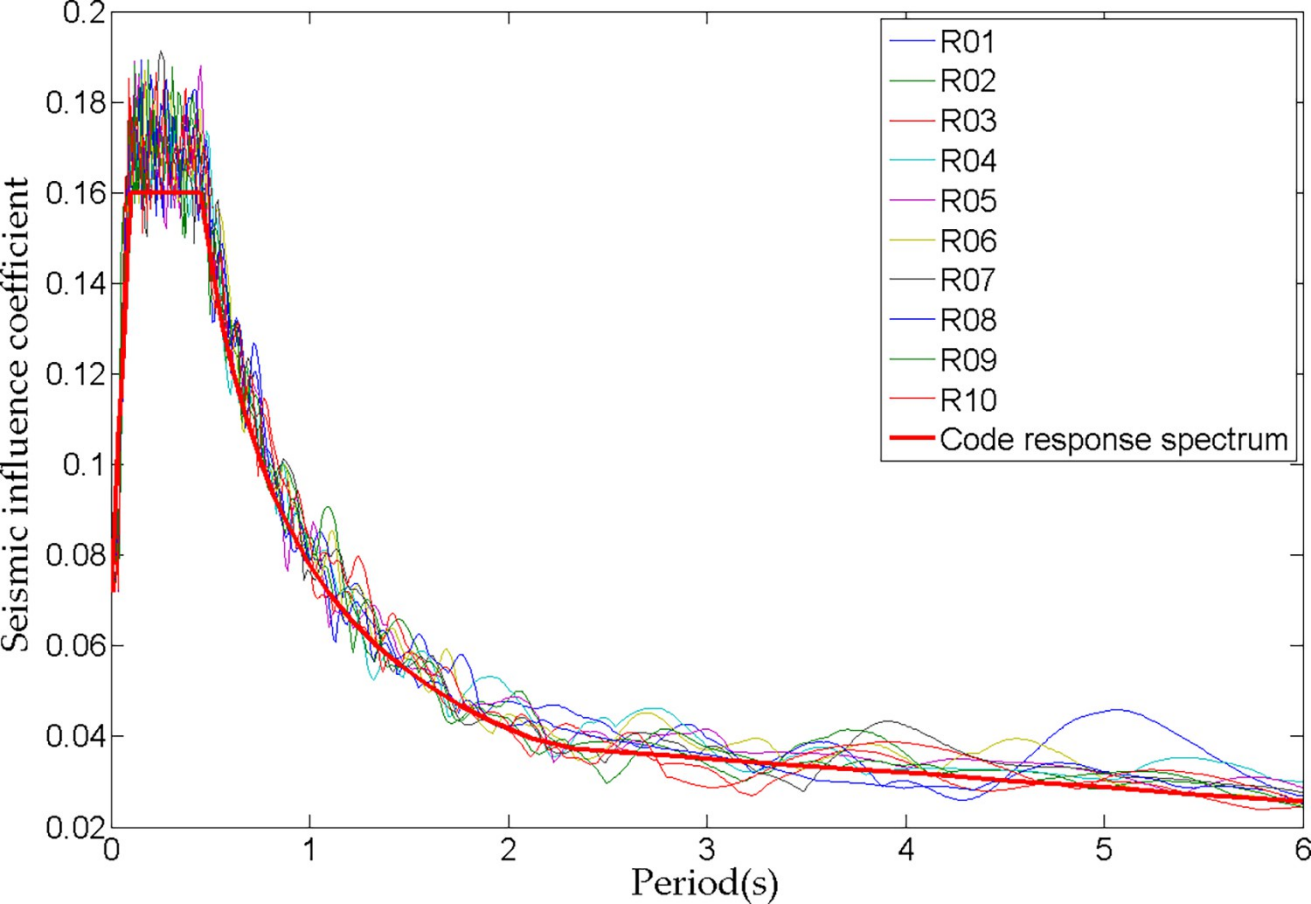
Response spectrum curves.

Since the discreteness of the seismic waves selected in the time course analysis will have a significant impact on the calculation results, 5 artificial seismic waves and 5 natural seismic waves with different phase characteristics (peak acceleration: 0.7 m/s^2^) were input in the X-axis direction under each working condition for calculation and statistical analysis in order to obtain more universal laws. The artificial seismic wave was generated by fitting the response spectrum in the China’s current Code for Seismic Design of Buildings [26]. Fig 7 illustrates that there is a good fitting effect between the seismic wave response spectrum and the response spectrum of the seismic design code.

## 6. Calculation results and discussion

### 6.1 Time difference

The time difference T_D_ of seismic wave input at different structural bearing points can be obtained by the following formula:

$$T_{D}=P_{S}/V_{S}=\sqrt{H^{2}+L^{2}}/V_{S}$$ (15)

Where P_S_ is the propagation distance of seismic waves, which is determined by the height H of the vertical part and the length L of the horizontal part in this model, considering the effect of both the horizontal and the vertical direction on P_S_. V_S_ refers to the shear wave velocity (SWV), whose value is determined by the soil layer type of the site where the structure is located.

**Fig 8:**
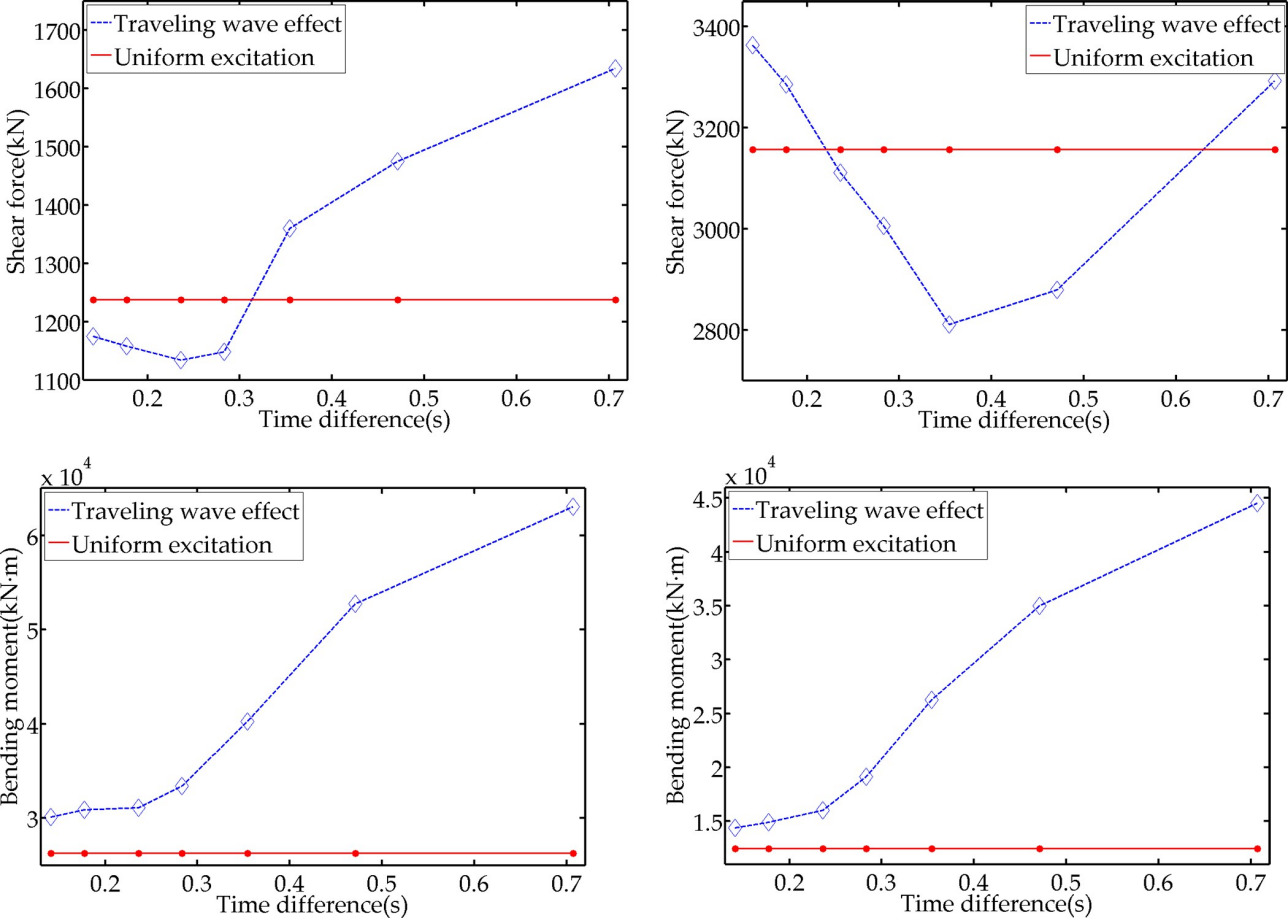
Variation diagram with time difference. (a) maximum shear force of vertical part; (b) maximum axial force of horizontal part; (c) maximum bending moment of vertical part; (d) maximum bending moment of horizontal part.

According to the China’s current Code for Seismic Design of Buildings, the SWV of common site types is generally between the range of 150 m/s and 800 m/s. The SWV selected in this study are 200 m/s, 300 m/s, 400 m/s, 500 m/s, 600 m/s, 800 m/s, and 1000 m/s, and the corresponding time differences calculated by Formula (15) are 0.707 s, 0.471 s, 0.354 s, 0.283 s, 0.236 s, 0.177 s, and 0.141 s. Then, substitute the time difference data obtained above into the seismic loads at the two bearings of the model to simulate the traveling wave effect, and obtain the seismic dynamic response under the traveling wave condition, followed by a comparison with the calculation results under the uniform excitation condition. The maximum shear force and bending moment of the vertical part’s control section and the maximum axial force and bending moment of the horizontal part’s control section are respectively extracted from the analysis results, and the average seismic response values of the 10 artificial seismic waves under different wave speed conditions are displayed in Fig 8.

Fig 8(A) suggests that with the increase of T_D_, the shear force of the vertical part under the traveling wave effect decreases first and then increases, while Fig 8(B) suggests that the axial force of the horizontal part increases first, then decreases, and gradually increases. Fig 8(C) and 8(D) illustrate that the structural bending moment increases as T_D_ rises under the traveling wave effect. By calculating more T_D_ conditions, the traveling wave effect response of the vertical shear force exceeds the uniform excitation response when T_D_ is greater than 0.3 s, and the traveling wave effect response of the horizontal axial force exceeds the uniform excitation response when T_D_ is less than 0.2 s or greater than 0.65 s.

### 6.2 Mass ratio

Given the input time difference of seismic wave, the mass parameters in the model can be adjusted to calculate the structure’s seismic dynamic response under the condition of different Гm values, followed by the analysis of the influence of the mass ratio on the traveling wave effect.

**Fig 9:**
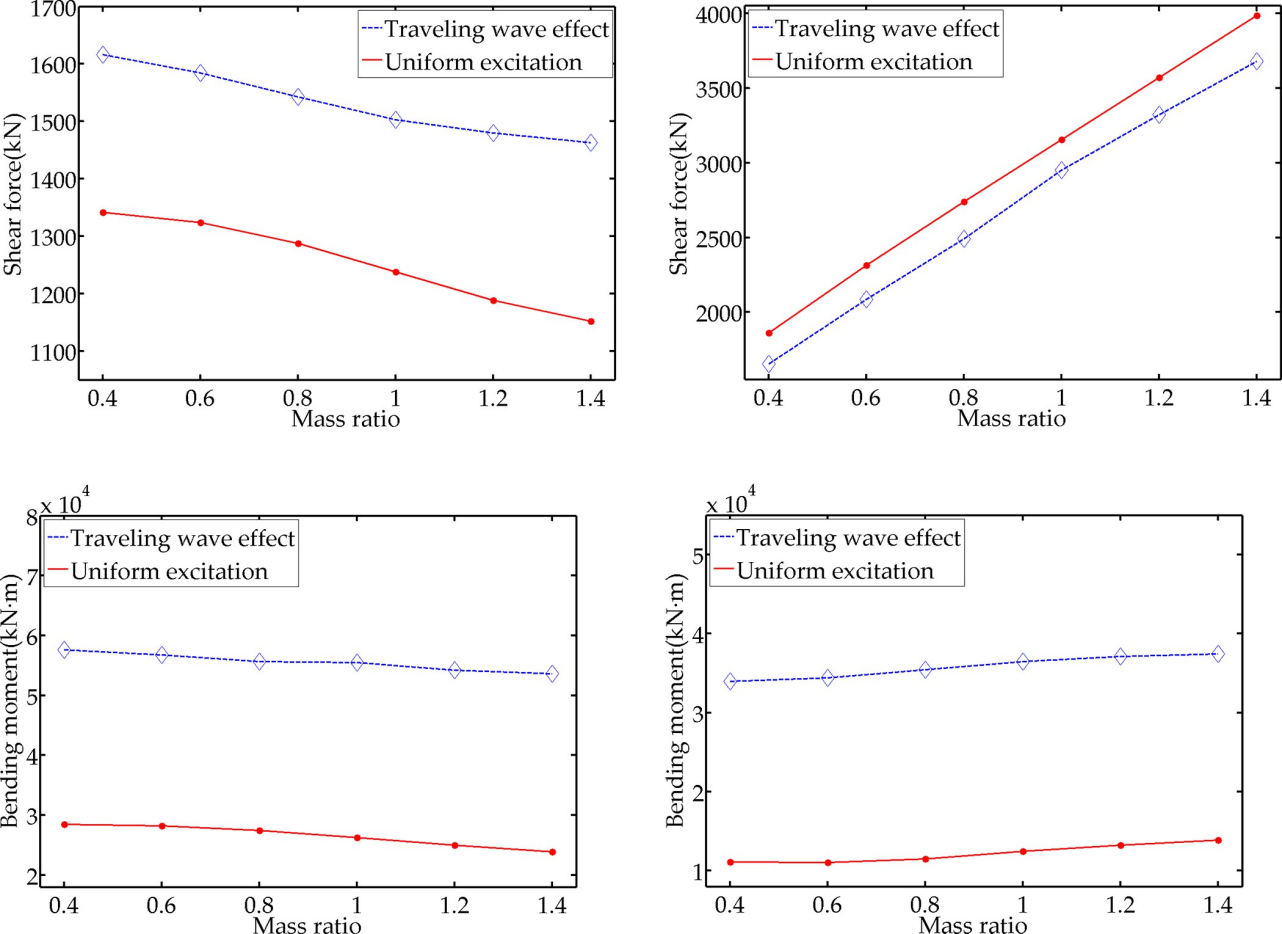
Variation diagram with mass ratio. (a) maximum shear force of vertical part; (b) maximum axial force of horizontal part; (c) maximum bending moment of vertical part; (d) maximum bending moment of horizontal part.

The external load input time difference is set as 0.5 s, and Г_m_ is set as 0.4, 0.6, 0.8, 1.0, 1.2, and 1.4. The mass of the model’s vertical part is unchanged. Thus, Г_m_ is reflected by adjusting the mass of the horizontal part. The remaining parameters remain unchanged. Fig 9 demonstrate the average value of seismic response of the 10 artificial seismic waves under different Г_m_ working conditions.

According to Fig 9, with the increase of Г_m_, the shear force and the bending moment of the vertical part gradually decrease under the two working conditions, while the axial force and bending moment of the horizontal part gradually increase. However, the difference between the traveling wave effect response and the uniform excitation response does not change significantly. Taking the difference when Г_m_ = 0.4 as the reference value, the maximum differences of the shear force and bending moment in the vertical part are 7.83% and 8.14%, respectively; the maximum differences of the axial force and bending moment in the horizontal part are 6.84% and 8.66%, respectively. This result reveals that the change in the mass ratio between the two parts of the structure will affect the absolute value of the seismic dynamic response, but not the difference between the traveling wave effect response and the uniform excitation response.

### 6.3 Stiffness ratio

Similarly, given the input time difference of seismic wave, the stiffness parameters in the model can be adjusted to calculate the structure’s seismic dynamic response under the condition of different Г_k_ values, followed by the analysis of the influence of the stiffness ratio on the traveling wave effect.

**Fig 10:**
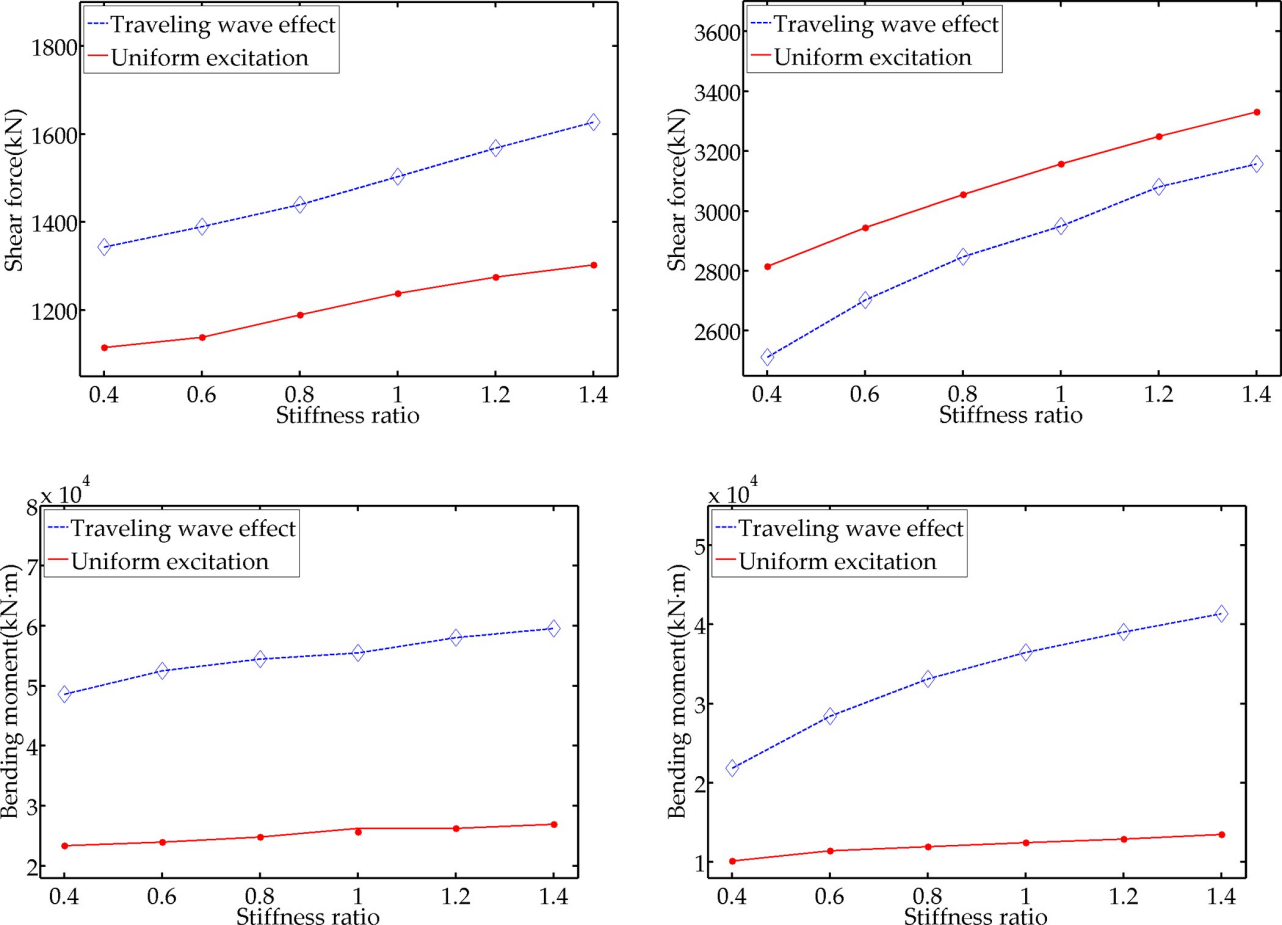
Variation diagram with stiffness ratio. (a) Maximum shear force of vertical part; (b) Maximum axial force of horizontal part; (c) Maximum bending moment of vertical part; (d) Maximum bending moment of horizontal part.

The external load input time difference is set as 0.5 s, and Г_k_ is set as 0.4, 0.6, 0.8, 1.0, 1.2, and 1.4. The stiffness of the model’s vertical part is unchanged. Thus, Г_k_ is reflected by adjusting the stiffness of the horizontal part. The remaining parameters remain unchanged. Fig 10 demonstrate the average value of seismic response of the 10 artificial seismic waves under different Г_k_ working conditions.

According to Fig 10, with the increase of Г_k_, the shear force, axial force, and the bending moment of the structure increase gradually under the two working conditions, and the difference between the traveling wave effect response and the uniform excitation response presents a significant change. Taking the difference when Г_k_ = 0.4 as the reference value, the maximum differences of the shear force and bending moment in the vertical part are 41.48% and 20.27%, respectively; the maximum differences of the axial force and bending moment in the horizontal part are 20.86% and 138.26%, respectively. This result reveals that the change in the stiffness ratio of the structure’s two parts will have an impact on the absolute value of the seismic dynamic response as well as the difference between the traveling wave effect response and the uniform excitation response.

## 7. Conclusions

To fill the research gap of the influence of traveling wave effect on the special Γ-type combined structure, an equivalent simplified model was creatively established in this paper to study this issue. The general law of the dynamic response of the model affected by traveling wave effect is summarized in three aspects through the time course analysis of the model for the subsequent research and practical engineering application of this type of structure.

1. The equivalent simplified model established by two kinds of models according to the characteristics of the Γ-type combined structure can simulate the original structure effectively.
2. As the time difference of seismic wave input at different supports increases, the bending moment of both parts of the Γ-type combined structure gradually increases. The shear force of vertical part first decreases and then increases, while the axial force of horizontal part first increases, then decreases and then gradually increases.
3. For the Γ-type combined structure, the traveling wave effect response is not always stronger than the uniform excitation response. Within a certain range of time difference, it will be weaker than the uniform excitation response.
4. The change in the mass ratio between the two parts of the Γ-type combined structure has basically no effect on the difference between the traveling wave effect response and the uniform excitation response, but the change in the stiffness ratio has a significant effect on this difference.
5. In the future design of lateral resisting system of Γ-type combined structure, the influence of traveling wave effect can be effectively reduced by changing the stiffness ratio between two parts of the structure.

## Supporting information

S1 Data (XLSX)
